# **Videothermometry to evaluate metabolic activity in real time during pneumectomy in rats** ^1^

**DOI:** 10.1590/s0102-865020190030000002

**Published:** 2019-03-21

**Authors:** Leonardo Waldstein de Moura Vidal, Paula Gebe Abreu Cabral, Marcelo Borges dos Santos, Fernanda Antunes, Matheus Roberto da Mota, Tomas Ottoni Barroso da Silva, Guilherme Alexandre Soares Monteiro, Jussara Peters Scheffer, Mariana da Silva Ribeiro, André Lacerda de Abreu Oliveira

**Affiliations:** I Fellow Master degree, Postgraduate Program in Animal Science, Animal Experimentation Unit (UEA), Universidade Estadual do Norte Fluminense (UENF), Rio de Janeiro-RJ, Brazil. Scientific, intellectual, conception and design of the study; acquisition, analysis and interpretation of data; technical procedures; manuscript preparation and writing, critical revision.; II MSc, UEA, UENF, Rio de Janeiro-RJ, Brazil. Scientific and intellectual content of the study; acquisition, analysis and interpretation of data; technical procedures; manuscript preparation.; III MSc, UEA, UENF, Rio de Janeiro-RJ, Brazil. Scientific and intellectual content of the study, manuscript preparation and writing.; IV PhD, Associate Professor, UEA, UENF, Rio de Janeiro-RJ, Brazil. Scientific and intellectual content of the study, statistics analysis.; V MSc, UEA, UENF, Rio de Janeiro-RJ, Brazil. Scientific and intellectual content of the study, technical procedures.; VI MSc, UEA, UENF, Rio de Janeiro-RJ, Brazil. Scientific and intellectual content of the study, manuscript preparation.; VII PhD, UEA, UENF, Rio de Janeiro-RJ, Brazil. Scientific and intellectual content of the study, technical procedures.; VIII PhD, Associate Professor, UEA, UENF, Rio de Janeiro-RJ, Brazil. Scientific and intellectual content of the study, critical revision, final approval.

**Keywords:** Metabolism, Thermometry, Rats, Wistar

## Abstract

**Purpose:**

To evaluate, in rats, the open field videothermometry in real time while performing left pneumonectomy for early diagnosis of cardiopulmonary changes.

**Methods:**

Twelve non-specific pathogen-free Wistar rats were randomly allocated into two groups; pneumectomy group (GP) and sham surgery group (GS). Mean arterial pressure, videothermometry in real time, of the right lung, and histopathological analysis of the remaining lung were evaluated in all animals.

**Results:**

Videothermometry in real time allowed identification of temperature variance of right lung after pneumectomy, indicating a significant decrease in temperature during evaluation. There was a statistical difference between M0 and M1, M1 and M2 and M0 and M2 (p<0.004) in GS, and significant difference between M0 and M1, M1 and M2, and M2 and M0 with p<0.0001 in GP.

**Conclusions:**

Left pneumonectomy in rats shows initial histopathological changes after 60 minutes of its completion, indicating a possible compensation beginning. The open-field videothermometry in real time proved to be efficient identifying the temperature changes of the remaining lung.

## Introduction

 Several complications, such as high mortality indices, can occur due to pneumonectomies. Mortality rates can be as much as 30% and in order to reduce this problem, complementary exams that are able to measure organs microcirculation during surgical procedures, are needed^1^. 

 An alternative to prematurely detect complications is the use of infrared thermometry. This technology is based on detection of temperature gradient that changes depending of the activity of certain tissue, which makes it a good alternative to detect microcirculation changes during surgical procedures. As already stablished, metabolic activity produces heat, and this is proportional to the intensity of chemical and physical reactions done on that tissue^2^.

 Infrared thermometry has already been used to identified body temperature of newborns, sites of inflammation, increases or decreases of temperature in trauma patients, and even to detect important diseases in animals such as lameness. Therefore changes in microcirculation will increase local heat and can be detected by these technology^3^ ^-^ ^5^.

 Another advantage is that this technology is harmless to the patient or surgical team, once it does not emit ionizing radiation and has a better sensitivity and specificity when compared to other imaging methods in order to identify metabolic activity in real time^6^.

 The present study aimed to evaluate the efficiency of this technology, during lung`s surgical procedures, and possibly decrease morbidity and mortality of patients. Besides it could be a great tool to early identify cardiopulmonary lesions during left pneumonectomy. 

## Methods

 The project was approved by the Ethics Committee for Animal Use, Universidade Estadual do Norte Fluminense (UENF), according to the Federal Law 11794/08 under the protocol number: 330750.

 Twelve non-specific pathogen-free Wistar rats weighting 300 g were included in the study and randomized into two groups: Six rats were submitted to pneumonectomy and six rats were submitted to SHAM surgery (Control group - GS). All animals were monitored during surgical procedures, data were recorded before pneumectomy (M0), 30 minutes after pneumectomy (M1) and 60 minutes after pneumectomy (M2). All animals were euthanized 60 minutes after assessment.

 The animals were sedated and anesthetized with a intraperitoneal injection (IP) of solution containing ketamine hydrochloride (100 mg.kg^-1^) and xylazine hydrochloride (5 mg.kg^-1^). Then, the carotid artery was isolated and cannulated with a 24-gauge intravenous silicone catheter (0.511 mm), filled with heparinized^1^ 0.9% saline solution (50 IU ml^-1^). The catheter was coupled to the BioAmp equipment sensor, responsible for coding blood pressure information and signal amplification in the form of computer graphics, where the data of invasive blood pressure and electrocardiographic tracing were recorded and saved by Labchart Pro 7.3.4 program.

 A tracheostomy tube was introduced into the trachea, and then mechanical ventilation (MV) was initiated by controlled volume through an artificial ventilation system (Harvard Rodent Ventilator model 683), at the respiratory rate of 80 cycles per minute and initial tidal volume of 10 ml.kg^-1^.

 The rats were submitted to median sternotomy and exposure of the thoracic organs with the aid of a *Weitlaner* retractor. The inferior pulmonary ligament was sectioned, structures were ligated and pneumonectomy was performed. After ligation of the left lung, the tidal volume was decreased to 8ml.kg^-1^.

 A transoperative videothermometry started in moment zero (M0) when MART (Metabolic Activity in Real Time) station was positioned above the animal at a height of 1 meter. This distance was essential to frame the entire animal in its field of valuation. The procedure was performed in 60 minutes and, subsequently, the medial lobe of the right lung and the heart were selected to evaluate the temperature in relation to procedure time using the MART station software 1.0^™^. 

 Animals of the control group (Sham) underwent sternotomy, as described above. The lung was manipulated similarly to the other group and analyzed for an hour. Then, animals were exsanguinated via an intra-arterial catheter until systolic blood pressure reached values below 20 mmHg and ECG showed a disruption of cardiac activity.

 The right lung was maintained inflated through tracheal ligature. After that, it was removed and fixed in 10% neutral-buffered formalin, submerged for 24 hours and sent for histological examination, where histological sections were stained with hematoxylin / eosin (HE).

 Statistical analysis was performed with GraphPad 6.0 (2014). The data were analyzed by bidirectional ANOVA, with a subsequent Duncan or Kruskal-Wallis mean test, depending on each case, respecting a p < 0.05 (99.95% reliability). For analysis of the qualitative variables, the Mann - Whitney tests were performed in order to produce multiple comparisons (2 to 2) between the groups; Friedman test for analysis, within each group, from time to time, in relation to the data obtained comparing with other variables, and Wilcoxon, making multiple comparisons (2 to 2), within each group, in relation to the data obtained with other variables.

 The histological results were analyzed through median of scores (descriptive statistics), by the degree of severity of the lesions found (congestion, multifocal hyperinflation, focal hyperinflation, focal hemorrhage), according to the evaluation of an independent observer. The scores were attributed following the estimated percentage of lesions appearance in the observed field for GS and GP, as shown in Table 1.

**Table 1:** Assessment of number of lesions measured by scores.

Number of lesions	Classification	Score
None		0
Slight	+	1
Moderate	++	2
Severe	+++	3

## Results

**Figure 1:**
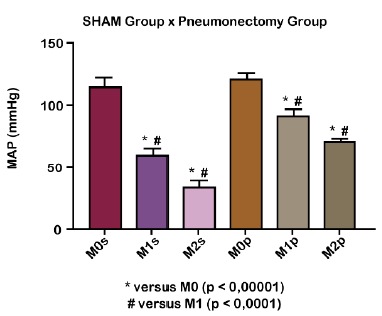
Decreased of mean arterial pressure (mmHg) of Wistar rats from the SHAM (GS) group and pneumectomy group (GP) at different times of the experiment. Before pneumectomy of GS (M0s); 30 minutes after pneumectomy of GS (M1s); 60 minutes of pneumectomy of GS (M2s); Before pneumectomy of GP (M0p); 30 minutes after pneumectomy of GP (M1p); 60 minutes after pneumectomy of GP (M2p).

 Firstly, the mean arterial pressure (mmHg) was evaluated, there was a statistical difference between M0 and M1, M1 and M2 and M0 and M2, in both groups, with p<0.0001 (Fig. 1).

**Figure 2:**
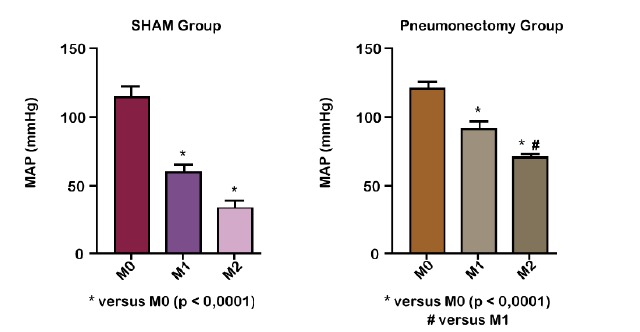
Comparison between mean arterial pressure (mmHg) of SHAM and pneumonectomy groups in Wistar rats at different times of the experiment. Before pneumectomy (M0); 30 minutes after pneumectomy (M1); 60 minutes after pneumectomy (M2).

 When compared between groups, there were statistical differences between M1s (M1 of SHAM group) and M1p (M1 of pneumectomy group) and M2s (M2 of SHAM group) and M2p (M2 of pneumectomy group), with p < 0.001 with higher values in GP (Fig. 2).

**Figure 3:**
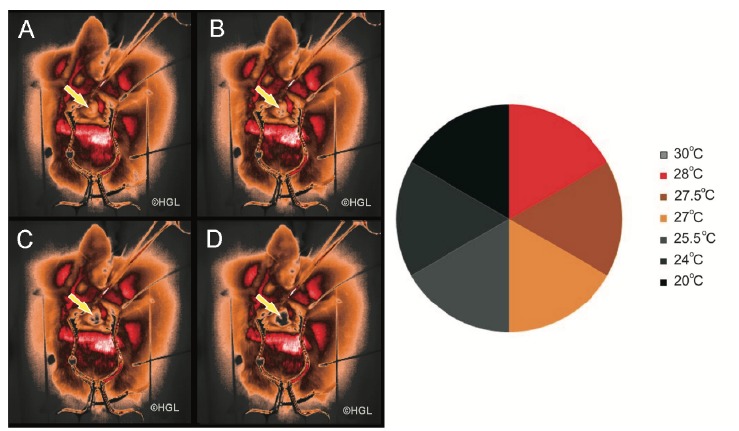
Temperature variation of the right lung evidencing heat loss (yellow arrows). Note that right lung assumes different color patches over period (**A, B, C, D**) that indicates heat loss (Fonte: MART Project -^©^HGL).

 During the whole experiment, when the gray isotherm was analyzed, it was possible to observe the temperature variation of the right lung after the left pneumonectomy, indicating a decrease in temperature during the moments of the experiment (Fig. 3).

**Figure 4:**
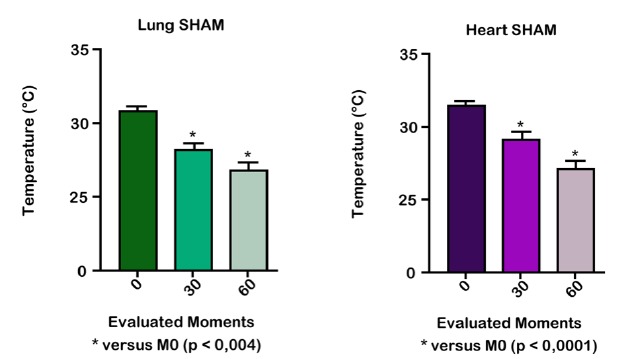
Variation in the right lung and heart temperatures of the SHAM (GS) group of Wistar rats at different times of the experiment. Reduction of temperature at moments M1 and M2.

 According to the evaluation of the right lung temperature in GS, there was a statistical difference between M0 and M1, M1 and M2 and M0 and M2 (p<0.004). The same was observed with heart temperatures, with p <0.0001 (Fig. 4).

 In the pneumonectomy group (GP), there was a significant difference in the right lung temperature between M0 and M1, M1 and M2, and M2 and M0. The same was observed with heart temperatures, with p <0.0001.

**Figure 5:**
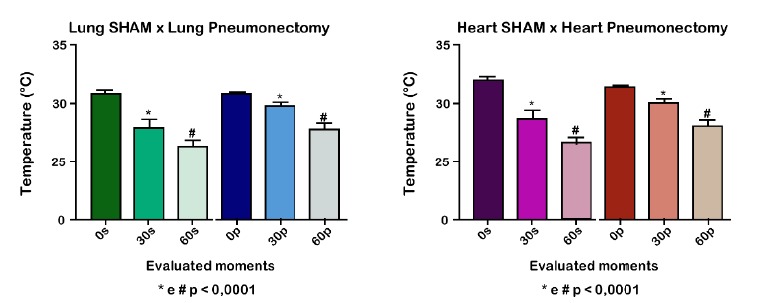
Variation of the right lung temperatures of SHAM (GS) and pneumonectomy (GP) groups in wistar rats at different times of the experiment. Reduction of the temperature in M1 and M2 of the SHAM group in relation to the pneumonectomy group.

 In a comparison between groups (GS and GP), there was a significant difference between right lung and heart temperatures in relation to M1 and M2, with p 0.001 (M1) and moments 2 (M2), with p <0.0001 (Fig. 5).

**Figure 6:**
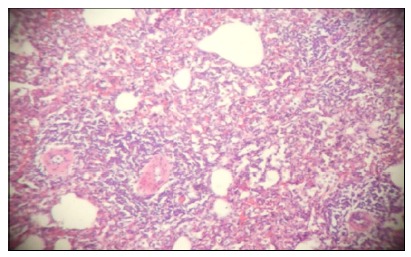
Photomicrograph of the lung - moderate to severe interstitial inflammatory reaction. Coloring in HE, x40.

 In all animals of GS there was moderate to severe inflammatory reactions, with few areas of focal atelectasis (Fig. 6).

 Similarly, an increase in cellularity was observed in all the animals of the pneumonectomy group, with a marked presence of neutrophils in interstitial infiltrate, denoting a moderate to severe inflammatory process. However, according to a median analysis of the quantitative evaluation of lesions in the remaining lung, areas of severe congestion, moderate focal hyperinflation and areas with a mild degree related to multifocal hyperinflation and hemorrhage were observed (Figs. 6 to [Fig f7] [Fig f8] 9).

**Figure 7 f7:**
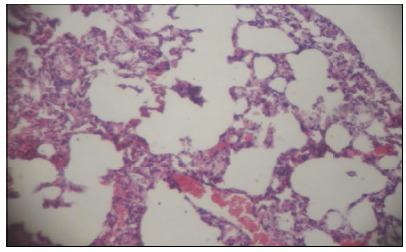
Photomicrography of rat lung from the experimental group - hyperinflation and hemorrhage. Coloring in HE, x100.

**Figure 8 f8:**
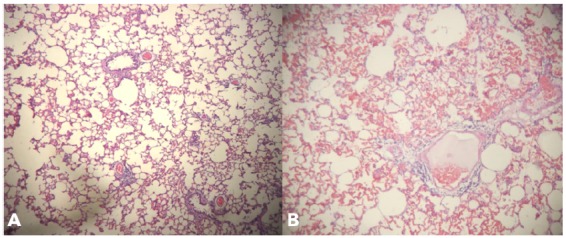
Photomicrography of the rat lung of the experimental group - intra-alveolar congestion and hemorrhage. Coloring in HE, x40 (**A** and **B**).

**Figure 9:**
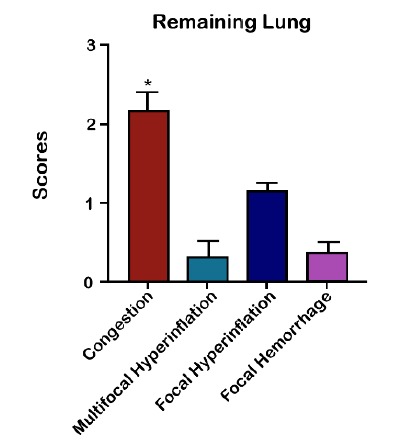
Median of the quantitative evaluation of pulmonary lesions in Wistar rats submitted to left pneumonectomy.

## Discussion

 The left side was chosen to the pneumectomy, due to the fact that the physiological changes that occur after a pneumonectomy are not well understood in all aspects, and may be related to the side that will be performed pneumonectomy^7^, and the complications on the right side have been higher, this may be due to the greater pulmonary area on the right side.

 There was a decrease in mean arterial pressure, which could be explained by the decrease in tissue temperature, which promotes vasodilation and, consequently, hypotension due to inefficiency of the smooth muscle of the vessel wall. We can still infer that although the ketamine does not depress the circulatory system, the xylazine has a vasodilatory effect, because it reduces muscle tone. Therefore, the decrease in blood pressure in both groups may be the result of the anesthetic drugs used, as well as the decrease in the animal temperature caused by thoracotomy and exposure of the organs to the external temperature of the operating room.

 Throughout the experiment, it was possible to observe the temperature variations through transoperative videothermometry, allowing monitor the variation of heat in both the right lung and the heart. In both groups, heart temperature was higher than lungs temperature throughout the experiment. This could be explained by the intensity of cardiac activity, and by the large amount of convection heat exchanges between blood flow of vessels and cardiac muscle.

 There was a significant decrease of temperature in M1 and M2 in both groups (GS and GP), when compared to moment 0 (M0). Anesthesia decreases temperature by decreasing the metabolic function and the muscular activity as well as affects arterial pressure^8^ ^,^ ^9^, promoting reduction of cardiovascular system efficiency and respiratory center^9^. In addition, the temperature of the operating room; The time of exposure to environments with low temperatures and body weight are factors of great influence in the drop of temperature^10^ ^,^ ^11^. 

 Although the temperature variation in the pneumonectomy group, this reduction was less intense compared to the SHAM group, which can be explained by the hyperflow resulting from the reduction of the total lung area. According to Brioschi *et al.* ^12^., a high emission of heat through the tissue indicates an increase in blood flow, while a decreased emission indicates hypoperfusion. Waller *et al.* ^13^ reports that the linear velocity of blood in the pulmonary microcirculation increases in pneumonectomies. This may contribute to an increase in temperature of the remaining lung, since all the cardiac output will be redirected only to the right lung, with an area deficit of 35%. In rats, the left lung corresponds to 35% of total lung mass.

 In addition, we could assume that due to the low arterial saturation caused by the reduction of hematose area, both the heart and the remaining lung need to increase their activity due to baroreceptor stimuli, with more energy consumption to maintain their functions. This may explain the less abrupt drop in heart and lung temperatures in the pneumonectomy (GP) group. Despite these changes, pneumonectomies stimulate the compensatory growth of the remaining pulmonary lobes, and some physiological factors have been proposed to regulate the beginning and modulation of compensatory pulmonary growth, including the mechanical distention of the remaining lung mass and the increase of blood flow to the residual tissue^14^.

 The histopathological analysis of both groups showed the presence of neutrophils, demonstrating that rats presented moderate to severe inflammatory reaction, which was an indirect sign of increase in vascular permeability due to endothelial lesion. Severe areas of congestion, moderate areas of focal hyperinflation and discrete areas of multifocal hyperinflation, as well as areas of focal hemorrhage were found pneumonectomy group. In this way, it can be observed that there was a structural change in the remaining lung during 60 minutes of evaluation. These results could represent the beginning of compensatory pulmonary growth. It has been reported on the appearance of new septa and thus the formation of new alveoli in the lung remaining after pneumonectomy^1^ ^,^ ^15^. 

 It is known that the compensatory growth of the remaining lung begins immediately after the operation, remains stable in the first 48 hours, and the whole lung mass is then restored in approximately 14 days^16^. However, the adaptation of the small vessels due to pressure changes caused by pneumonectomy is unknown and may be related to the genesis of pulmonary edema, as the increase in pulmonary vascular resistance causes stress on the endothelium and rupture of the cellular junctions, causing extravasation of fluid to interstitium.

 Furthermore its already know that, in healthy organisms, a temperature difference bigger than 0.2ºC should not occur, and if a variation of 0.3ºC is seen, a metabolic abnormality should be considered, based on the fact that central temperature is constant in homeothermic animals, such as rats. Besides this fact is already standardized that variations of 1ºC or bigger is a strong indication of body disfunction or better described as metabolic abnormality^17^. Therefore by analyzing Figure 5, its possible to infer that temperature difference bigger than 0.2ºC occurred in GP when compared to GS, in the remaining lung and also in the heart, which corroborates to the fact that videothermometry can predict early metabolic changes, as seen in histopathological analyses, which showed differences between groups in present work.

## Conclusion

 Real-time videothermometry was efficient in identifying the changes in temperature of the remaining lung and heart, allowing its use in the trans-operative as a method capable to predict important alterations, thus enabling the early correction of these changes.
